# Comprehensive analysis of structural variants in chickens using PacBio sequencing

**DOI:** 10.3389/fgene.2022.971588

**Published:** 2022-10-20

**Authors:** Jinxin Zhang, Changsheng Nie, Xinghua Li, Xiurong Zhao, Yaxiong Jia, Jianlin Han, Yu Chen, Liang Wang, Xueze Lv, Weifang Yang, Kaiyang Li, Jianwei Zhang, Zhonghua Ning, Haigang Bao, Chunjiang Zhao, Junying Li, Lujiang Qu

**Affiliations:** ^1^ Department of Animal Genetics and Breeding, National Engineering Laboratory for Animal Breeding, College of Animal Science and Technology, China Agricultural University, Beijing, China; ^2^ Institute of Animal Sciences, Chinese Academy of Agricultural Sciences, Beijing, China; ^3^ Beijing Municipal General Station of Animal Science, Beijing, China

**Keywords:** chicken, structural variants, whole-genome sequencing, pacbio data, genetic diversity

## Abstract

Structural variants (SVs) are one of the main sources of genetic variants and have a greater impact on phenotype evolution, disease susceptibility, and environmental adaptations than single nucleotide polymorphisms (SNPs). However, SVs remain challenging to accurately type, with several detection methods showing different limitations. Here, we explored SVs from 10 different chickens using PacBio technology and detected 49,501 high-confidence SVs. The results showed that the PacBio long-read detected more SVs than Illumina short-read technology genomes owing to some SV sites on chromosomes, which are related to chicken growth and development. During chicken domestication, some SVs beneficial to the breed or without any effect on the genomic function of the breed were retained, whereas deleterious SVs were generally eliminated. This study could facilitate the analysis of the genetic characteristics of different chickens and provide a better understanding of their phenotypic characteristics at the SV level, based on the long-read sequencing method. This study enriches our knowledge of SVs in chickens and improves our understanding of chicken genomic diversity.

## Introduction

Structural variants (SVs) are rearrangements larger than 50 bp in chromosomes (Alkan et al., 2011). Several SVs have been associated with phenotypic variation (Wright et al., 2009; Imsland et al., 2012; Dorshorst et al., 2015; Guo et al., 2016; Chen et al., 2017; Bertolotti et al., 2020; Wu et al., 2021), productive traits (Liu et al., 2010; Zhao et al., 2016; Liu et al., 2019), immune function, and disease resistance (Luo et al., 2013; Bickhart and Liu, 2014) in animals.

Chicken is an important protein source for humans. According to the Food and Agricultural Organization (https://www.fao.org/home/en), between 2000 and 2020, the proportion of poultry meat traded on the international market has doubled. The poultry meat Index reached 130,39 points in June 2022, an increase of 28.9% more than last year. Therefore, genetic research on chicken is highly valuable. Previous studies have shown that structural variations in domestic chicken play a vital role. For example, the pea comb is caused by duplication of the first intron of the *SOX5* gene (Wright et al., 2009), and late feathering is caused by duplication of the K locus (Elferink et al., 2008). Although there is much evidence that SVs are important, they have been largely understudied in comparison to single-nucleotide variants (SNPs) because they can be difficult to detect. An SV likely remains unknown unless the sequence reads cover its entire length (Sedlazeck et al., 2018).

Substantial progress has been made in SV detection over the last decade using short-read sequencing data; however, the structural variation of some complex traits and repetitive regions remains unsolved (English et al., 2015; Chaisson et al., 2019). Nevertheless, long-read sequencing technology has introduced new possibilities for identifying complex variants. Pacific Biosystems (PacBio) is one of the leaders in this field, and its sequencers can generate reads over 10 kb, which may span the entire variation region (Merker et al., 2018; van Dijk et al., 2018). Studies have shown that long-read sequencing improves the detection accuracy and sensitivity of SVs (Kosugi et al., 2019; Mahmoud et al., 2019; Liu et al., 2020b).

Here, we took advantage of whole-genome re-sequencing data, including long- and short-read data, to investigate SVs in 10 chickens with different genetic backgrounds and significant phenotypic differences. We attempted to detect SVs related to chicken phenotypic traits and mined more phenotype-related candidate genes. These results may provide a reference for future research on structural variations in chickens.

## Materials and methods

### Sample collection and DNA extraction

A total of 10 chickens were collected from different breeds for genomic sequencing. This group consisted of four commercial chickens (White Leghorn, WL; Rhode Island Red, RIR; Cornish, COR; White Plymouth Rock, WR), five indigenous chickens (Silkies, SK; Beijing You, BY; Tibetan, TB; Piao, P; Dong Tao, DT), and one wild chicken (red jungle fowl, RJF). All samples were obtained from experimental farms, including WL, WR, TB, RIR, COR, SK, and BY from poultry genetic resources and breeding experimental bases of China Agricultural University, Piao and DT chicken from Yunnan and Vietnam, and RJF from Indonesia. (Supplementary Table S1; Supplementary Figure S1).

Blood samples were collected from the wing vein and stored at –20°C for DNA extraction. For PacBio continuous long read (CLR) sequencing and short-read sequencing (Illumina sequencing), genomic DNA was extracted using a TIANamp Blood DNA Kit DP348 (Tiangen Biotech Co. Ltd., Beijing, P.R. China).

### PacBio and illumina library construction and sequencing

For short-read sequencing, at least 3 μg of genomic DNA was used to construct a paired-end sequencing library with an insert size of approximately 350 bp, which was then sequenced on the Illumina HiSeq X Ten and HiSeq 2000 platforms (Illumina, San Diego, CA, United States) following the manufacturer’s instructions at Novogene. Clean reads were obtained by removing reads containing adapters, poly-N, and low-quality reads from raw data (average 10X coverage of the chicken genome).

For PacBio continuous long reads, libraries with an average insert size of 20 kb were constructed using the SMRTbell Template Prep Kit. PacBio sequencing was performed on the Pacific Bioscience Sequel II platform. Finally, Smrtlink was used to filter low-quality raw data (minLength = 50, minReadScore = 0.8).

### Alignment and structural variants detection

Different sequencing techniques were used for read alignment and SV calling. For short-read sequencing, the filtered reads were mapped onto the GRCg6a reference genome using the Burrows-Wheeler Aligner (BWA-V0.7.17) with default parameters (Li and Durbin, 2010). Mapping results were then converted into BAM format and sorted using SAMtools version 1.9 (Li et al., 2009). Duplicate reads were removed using Picard version 2.3 (https://broadinstitute.github.io/picard/). GATK-V4.1.9.0 (McKenna et al., 2010) (default settings) was then used to call the raw single nucleotide polymorphisms (SNPs).

A consensus-based approach was used to call the SVs, which involved parallel SV calls by Delly_v0.8.7 (Rausch et al., 2012) and Manta_v1.6.0 (Chen et al., 2016) to obtain a high-confidence set of SVs. In this study, we used the default parameters recommended by the software for structural variation detection. The final SVs were filtered using the following criteria: SVs passing the quality filters (flag PASS) with a length of ≥50 bp. Finally, SV events were merged into a VCF file using SURVIVOR-V0.1.7 for downstream analysis.

For long-read sequencing, Smrtlink was used to filter the low-quality raw data. Alignment of subreads to the GRCg6a chicken reference was undertaken using aligner NGMLR-V0.2.7 (Sedlazeck et al., 2018) and called for genome-wide SV using the SV-calling algorithms Sniffles-V1.0.12 and SVIM-V1.2.0 (Sedlazeck et al., 2018; Heller and Vingron, 2019). To reduce the number of false positives, a strict filtering protocol was followed.

**FIGURE 1:**
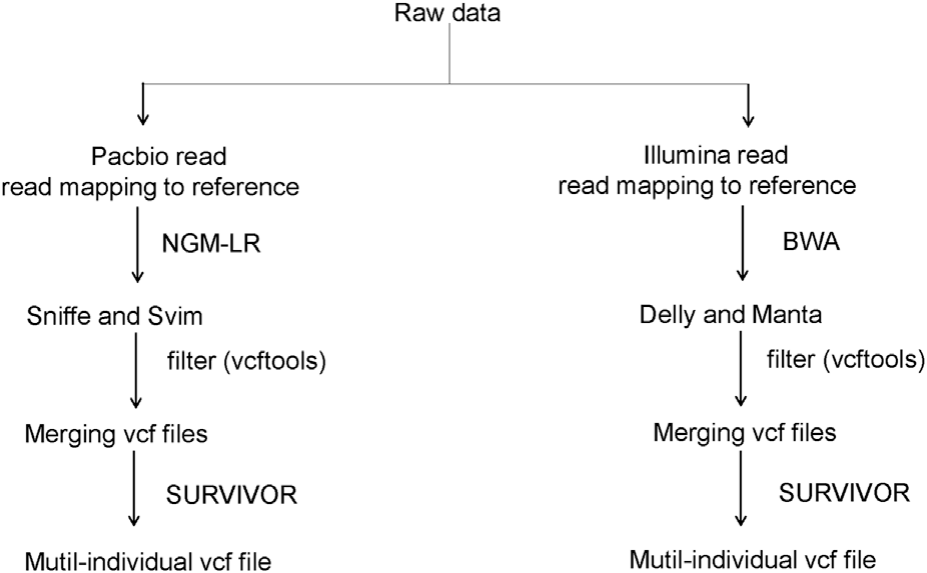
Overview of the workflow of this study.

Uncertain and low-quality SVs (flag: IMPRECISE/UNRESOLVED) were removed and only SV calls longer than 50 bp with more than four supporting reads were retained. In the same position, only the SVs that were most supported were retained. Finally, the SV events predicted for each individual were merged into a VCF file using SURVIVOR (Jeffares et al., 2017) for downstream analysis (Figure 1 illustrates the operation process). Intersection sets for SVs across each chicken were created using UpSetPlot (http://bioconductor.org/biocLite.R).

### Determination of structural variants hotspots

We divided the chicken genome into 2,117 non-overlapping 500 kb intervals. We then mapped 46,825 SVs into 2,117 intervals with four types of variations (INS, INV, DUP, and DEL). We assumed that the number of SVs mapped to each interval would follow a Poisson probability distribution if the SVs were distributed randomly across the genome. We generated an expected Poisson distribution, which was used to determine the criteria for SV hotspots. The intervals containing an empirical SV number equal to or higher than the 99th percentile of the expected Poisson probability distribution were classified as SV hotspots. The genes were annotated using the VEP of ENSEMBL (http://ensembl.org/Tools/VEP). The gene ontology (GO) enrichment analysis was conducted using DAVID (https://david.ncifcrf.gov/). Functional clustering included three aspects: molecular function (MF), cell component (CC), and biological progress (BP). The GO terms with an adjusted value smaller than 0.05 were considered significant.

### Genetic structure analysis

The obtained SV dataset was used to construct phylogenetic trees using the NJ method for the SNP data. VCF tools (v0.1.13) were used to convert VCF files to a PLINK format, and then in-house bash scripts were used to convert these into a PHYLIP format for input into IQTree (Nguyen et al., 2015). The tree was plotted using iTOL (https://itol.embl.de/). We performed a principal component analysis for the SNP genotypes and visualized the results in R using the package SNPrelate. (Zheng et al., 2012).

## Results

### Sequencing and structural variants detection

**FIGURE 2:**
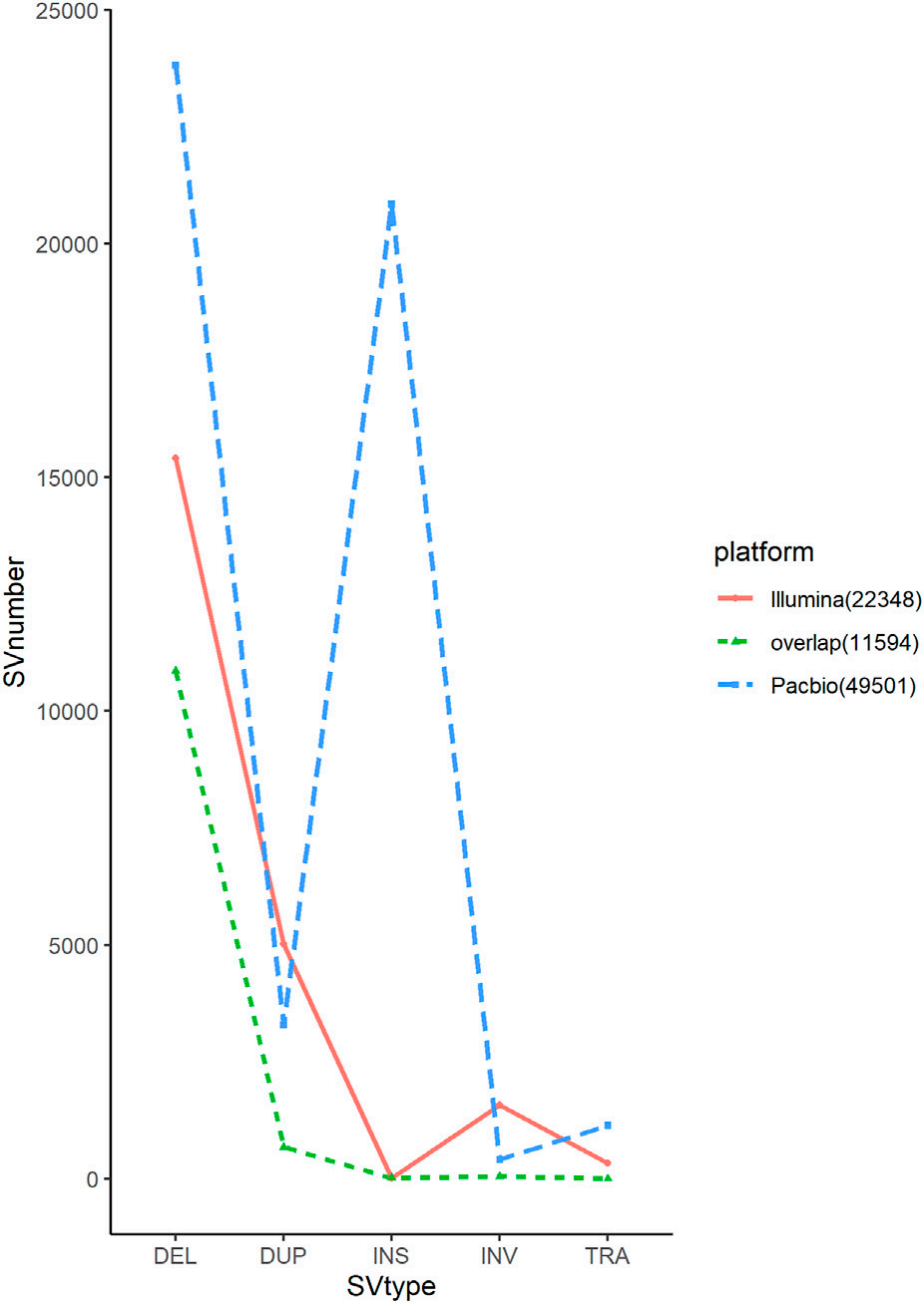
Comparison of structural variations in PacBio and Illumina sequencing platform.

In long-read sequencing, the average sequencing depth of 10 chickens of different breeds was approximately 39x, and in short-read sequencing, the average sequencing depth was approximately 19x (Table1). Our sequencing data with sufficient coverage ensured high-sensitivity SV calls. The SVs in this study were larger than 50 bases, and there was no limit for the maximum length. Long sequencing technology can cover substantial chromosomal regions and directly reveal long deletions, insertions, duplications, inversions, and translocations, allowing the detection of structural differences between genomes in each chicken. The 49,501 SVs were obtained only from PacBio data, representing 23,817 deletions, 3,292 duplications, 20,847 insertions, 407 inversions, and 1,138 translocations using two detection tools. For short-read sequencing, 22,348 SVs were detected. The proportion of deletions was the highest among the different SV types. In this study, long-read sequencing identified more SVs than short-read sequencing did. We found that the stability and reproducibility of SV results from the long-read sequencing software were better than those of SV results from the short-read sequencing software. The number of SVs detected by Sniffles and SVIM was similar, and the number of overlapping SVs was higher, whereas the number of structural variants detected by Delly and Manta was different, and the number of overlapping SVs was lower. We compared the results of the SVs generated by the two platforms to identify overlapping variants. The results showed that short-read and long-read sequencing overlapped 51.87% of the SVs (Table2; Figure 2).

**TABLE 1:** Summary of sequencing information for the 10 chicken samples.

Sample	Type	Sex	Pacbio data				Illumina data	
			Average Depth	Mean Reads	N50 for Raw reads	Mapping Ratio	Average Depth	Mapping Ratio
SK	indigenous chicken	F	41.14X	9.5 kb	15.54 kb	87.38%	45.87	99.13%
TB		F	25.44X	10.1 kb	11.4 kb	96.27%	13.73	99.12%
BY		F	52.87X	17.6 kb	20.5 kb	94.73%	14.04	99.12%
P		M	47.89X	13.4 kb	14.3 kb	94.36%	9.60	97.00%
DT		M	82.11X	17.9 kb	20.7 kb	94.42%	10.74	98.66%
Cor	Commercial chicken	F	23.71X	9.3 kb	10.4 kb	96.64%	12.53	99.21%
WR		F	22.40X	9.9 kb	11.2 kb	96.29%	12.72	99.14%
WL		F	41.23X	10.1 kb	15.55 kb	87.76%	48.04	99.29%
RIR		F	25.17X	10.7 kb	12.2 kb	96.29%	12.10	99.15%
RJF	Wild Breed	M	23.69X	6 kb	7.4 kb	93.1%	8.13	99.15%

Silkies, SK; tibetan, TB; beijing you, BY; piao, P; dong tao, DT; cornish, Cor; White Plymouth Rock, WR; white leghorn, WL; rhode island red, RIR; red jungle fowl, RJF.

**TABLE 2:** Number of SVs detected by Illumina short reads and PacBio long reads in 10 chicken samples.

Sample	Long-read sequencing SV number		Short-read sequencing SV number			
	sniffle	svim	final	delly	Manta	final
TB	12995	14136	11109	11003	20142	4472
BY	20160	21662	15127	11246	20984	4714
P	21155	23185	15080	8206	21339	2877
DT	24990	26585	17410	8113	23983	3161
SK	17357	19702	14823	19236	37169	7681
WL	15663	17331	13219	18275	33956	7507
Cor	11052	11829	9486	9136	17104	3733
RIR	12311	13306	10465	9550	17470	3862
WR	11253	12162	9668	10211	18708	4239
RJF	15665	16153	10238	6543	12168	2603
Total		49501			22348	

### Genome-wide structural variants distribution and structural variants hotspot analysis by PacBio data

**FIGURE 3:**
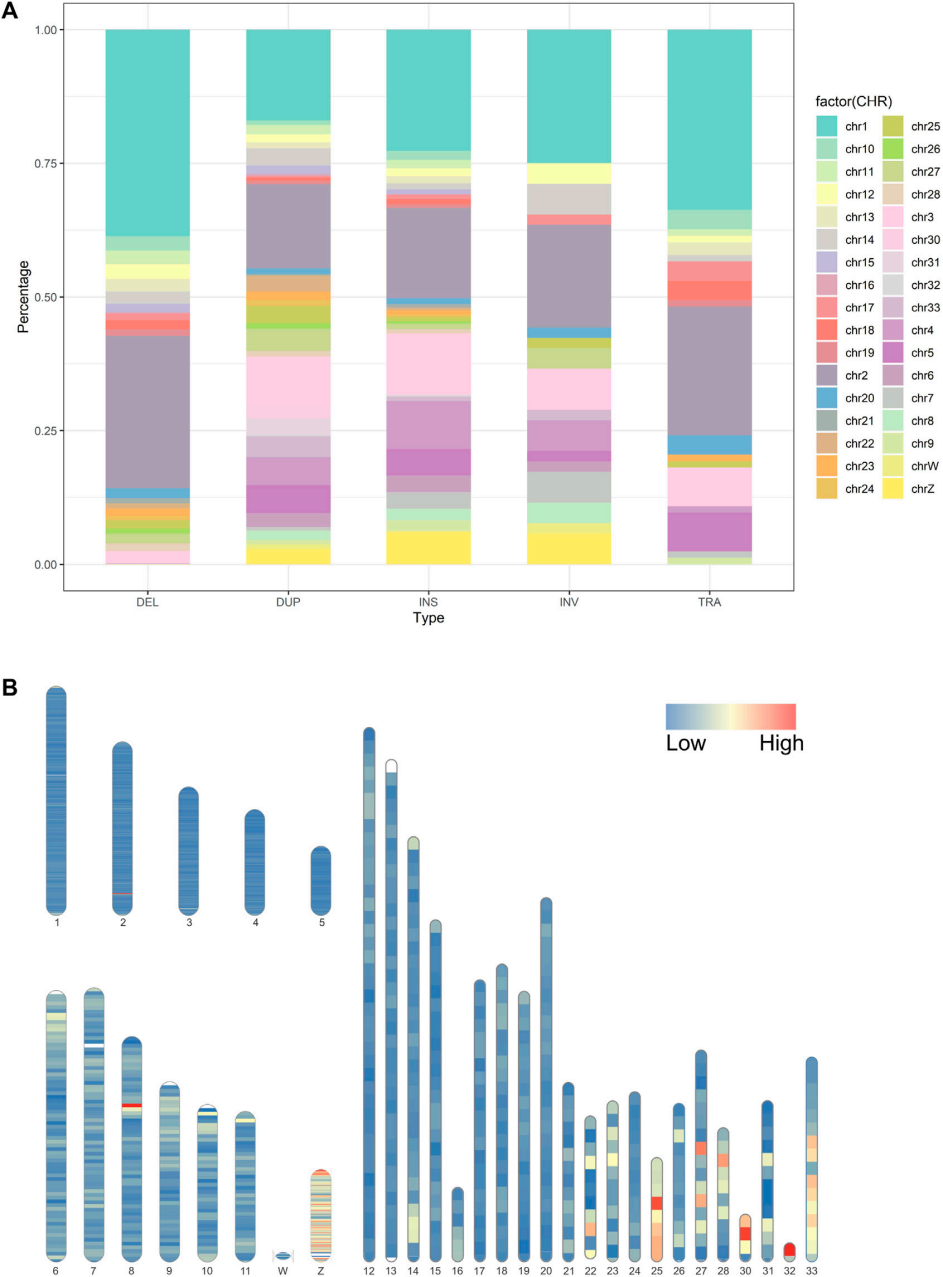
**(A).** Distribution of structural variations among the chicken chromosomes; **(B)**. Distribution of the SV density on chicken chromosome ideogram. This was drawn using Rideogram R package. Note: Macro-chromosome (1-5); Intermediate-chromosome (6-11); Micro-chromosome (12-28, 33); Sexual-chromosome (W, Z).

Genomic SVs are not evenly distributed on chromosomes (Perry et al., 2006; Gokcumen et al., 2011). Analyzing the bias of different types of SVs on chromosomes facilitates the understanding of the distribution characteristics of genetic variation and the impact of SVs on the genome. The longer the chromosome, the more SVs it contains (Figure 3A). For example, Chr1 to Chr5 account for 21.7%, 16.2%, 9.8%, 7.6%, and 4.3% of the total SVs, respectively. It is interesting to note that micro-chromosomes contain a greater density of variation than longer chromosomes. For instance, chromosomes 30 and 32 have a density of 1 SV every 2.94 kb (620 SVs in 1.8 Mb) and 2.06 kb (353 SVs in 0.7 Mb), respectively. For comparison, large chromosomes show much less structural variation (e.g. One every 20 Kb on chromosome 1); the detailed results are presented in Supplementary Table S2. The distribution of SVs had several hotspots on the chromosomes. We further investigated the size of the identified SVs across the chicken genomes, and the majority of smaller-sized SVs were less than 1 kb. Most inversions were ∼680 Mb, spanning the largest region among all types of SVs. To observe the distribution of all the SVs throughout the chicken genome, we used SVs to generate a genome-wide variation distribution map. An investigation of chicken genome SV density, and a better understanding of SV length and number chromosomal distribution. Figure 3B shows the density and distribution of all SVs we used for analysis on chicken chromosome ideogram. The SV density of micro-chromosomes is higher than that of large chromosomes in some regions.

**FIGURE 4:**
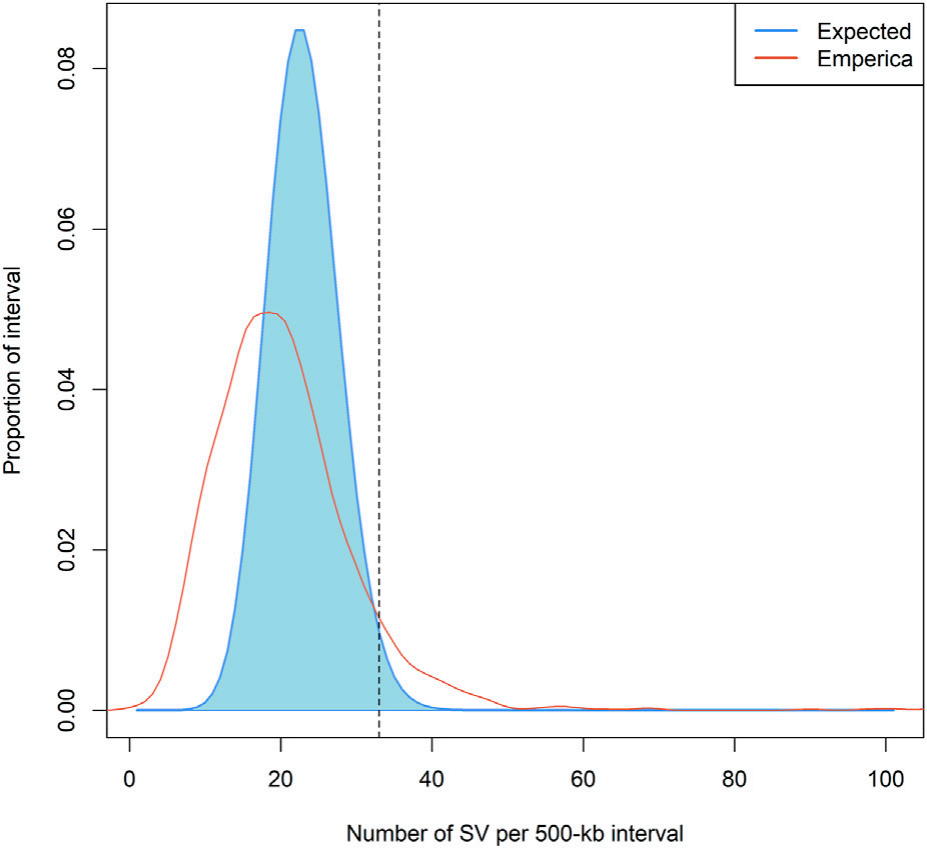
Non-random distribution of SVs across chicken genomes. Note: The density plot showed the empirical and the expected probability distributions of the number of SV (s) within a 500 kb long interval of the chicken reference genome (gal6). The empirical probability distribution was estimated by the actual number of SVs within each of the 2117 intervals. The expected probability distribution was estimated assuming a Poisson distribution.

We identified SV hotspots in the chicken genome by comparing the expected and empirical distributions of the SVs (Figure 4). A total of 113 intervals (interval size of 500 kb, 7% of all intervals) containing ≥33 SVs in the empirical distribution were characterized as SV hotspots. These intervals fall within the 99th percentile (or higher) of the expected distributions. All intervals included 6,180 SVs with 2,957 deletions, 791 duplications, 2,396 insertions, and 35 inversions. In total, 768 genes were identified in the defined SV hotspot regions. Among these, the largest number of SV sites (n = 517) was detected on chromosome 2. The SV hotspots are presented in Supplementary Table S3. To functionally infer the SV hotspot data for chickens, we conducted functional enrichment analysis for genes positioned within the SV hotspots. Gene enrichment and ontology analysis results showed that the genes were related to terms such as regulation of stem cell population maintenance, propanoate metabolism, cell proliferation, and calcium channel regulator activity (Supplementary Table S3).

## Special structural variants in chickens

**FIGURE 5:**
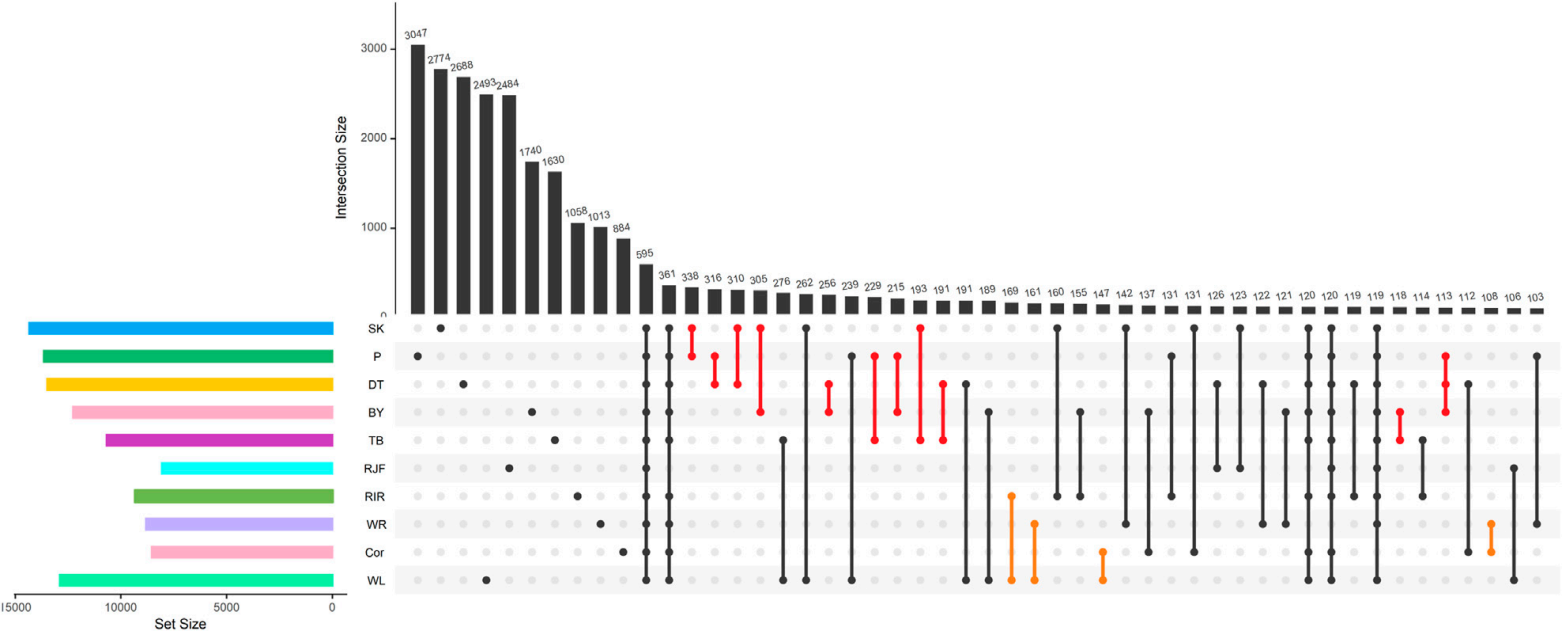
UpSet plot of SVs results detected in each chicken. The intersection between Asian chickens is represented by a vertical red line; the intersection between European chickens is represented by a vertical orange line, the others is represented by a vertical black line. Note: In the upper left, Venn diagram of the same genetic background. In the bottom left panel, horizontal bars represent the number of SVs detected by each chicken, vertical bars represent the size of SVs detected in each chicken, black dots represent the sample set, and the intersection between chickens is represented by a vertical black line.

To date, little is known about the SVs of some unique indigenous Chinese chickens. To further explore the relationship between these specific SVs and chicken phenotypes, we analyzed SVs in 10 chickens with different phenotypes. There were 654 SVs in all 10 individuals and 27,454 SVs were observed in only one individual. Among them, DT (5,186) and RJF (4,401) chickens had more specific SVs, and highly selective commercial chickens (COR, WR, RIR, and WL) had 2,522; 2,714; 3,224; and 5,207 specific SVs, respectively. A total of 3,559; 4,308; 2,349; and 3,570 specific SVs for BY, P, TB, and SK, respectively, were detected (Supplementary Table S4). These results showed that more intensive artificial selection resulted in a reduction in genetic variation. To investigate the common SVs among chickens, we analyzed the SVs numbers among chickens of similar backgrounds (Asian and European). Different chickens exhibit varying degrees of common SVs. We showed the intersections of ten chickens for SV numbers using an UpSet plot, where interactions were ranked by SV number. A total of 1023 intersections are included. In Asian chickens, 63 intersections share among themselves, while only 15 intersections share among themselves in European chickens (Supplementary Table S5). The first 50 intersections are shown in Figure 5. The plot showed that most SVs are private to each individual, followed by SVs shared in all chickens. It implies that the reference genome lacks portions relevant to most animals. Additionally, there is also a great deal of SVs (15/50) that are shared privately in Europe or Asian populations. There could be some phenotypic and growth differences in the chickens affected by these SVs (Supplementary Table S6). These SVs may affect the phenotypes and growth characteristics of chickens. Previous studies have shown that the *SOX5* gene is associated with pea comb in chickens (Wright et al., 2009). A *SOX5* mutation was found in the genome of Dongtao chickens, consistent with previous observations. A mutation in *SH3RF2* was found in WR chickens, which was previously reported to influence chicken growth. We also identified several MHC-related genes (*BZFP1*, *TAP1*, and *IL4I1*), all of which are located on chromosome 16. In addition, *AKAP8L*, *ASCC3*, *BG8*, *OPN5L1*, and *SH3RF2* are involved in the immune response. Several genes, including *AKAP8L*, *ASCC3*, *BG8*, *OPN5L1,* and *SH3RF2*, were also found to be involved in the immune response. It is possible that these SVs may affect indigenous chickens, resulting in stronger disease resistance than those in commercial chickens.

### Genetic structure of chickens

**FIGURE 6:**
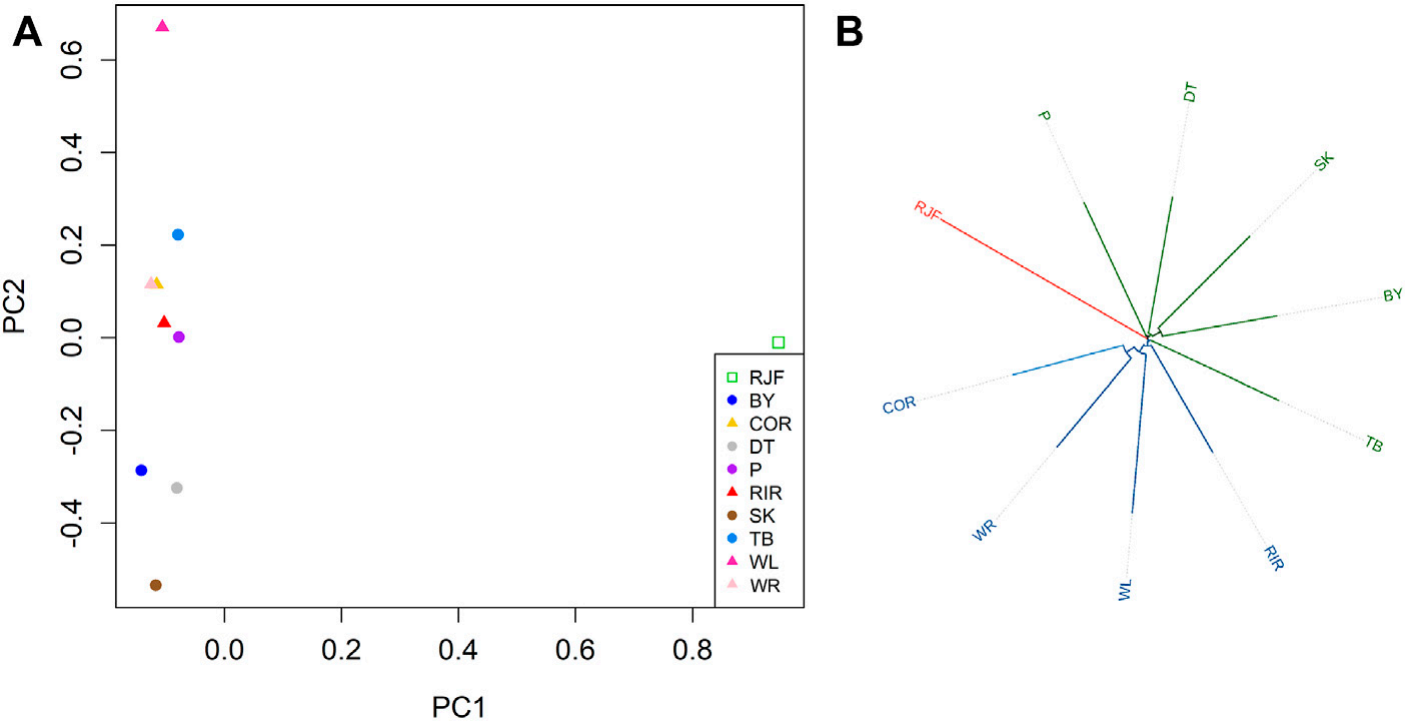
Genetic structure of SVs in different chickens. **(A)** PCA analyses; **(B)** Phylogenetic tree. Note: Silkies (SK); Tibetan (TB); Beijing You (BY); Piao (P); Dongtao (DT); Cornish (Cor); Plymouth Rock (WR); White Leghorn (WL); Rhode island Red (RIR); Red jungle fowl (RJF).

We used SVs to infer the genetic structure of all 10 chickens (Figure 6). PCA indicated that each chicken was distinctly different from the others. The first principal component (PC1) distinguished wild RJF chickens from domestic chickens. Commercial chickens were distributed in the upper half of the second principal component (PC2), while local chickens were mostly distributed in the lower half of PC2, indicating that indigenous chickens were genetically different from commercial chickens based on SVs (Figure 6A). We constructed phylogenetic trees for chickens (Figure 6B), where all chickens were divided into three major branches: red jungle fowl (RJF), commercial chickens (WR, WL, RIR, and COR), and indigenous chickens (TB, BY, SK, DT, and P). In brief, the phylogenetic tree and PCA analyses support the division of the panel into commercial chickens, indigenous chickens, and wild chickens, and the results of SVs could help in understanding their population genetics.

## Discussion

SVs have been recognized as important sources of genetic variation, and are the main contributors to phenotypic diversity and evolutionary adaptation in chickens (Wang and Byers, 2014; Seol et al., 2019; Fernandes et al., 2021). Compared with SNPs, SVs tend to cause changes in the gene structure, which could change their function and cluster within SV hotspots, which are beneficial to organisms during the evolution process (Liu et al., 2020a). In this study, we used long-read sequencing technology to detect SVs in 10 chickens, analyzed structural variation in the chicken genome, and identified several SVs that may be related to chicken growth and reproduction, which can provide further information for future studies.

PacBio sequencing appears to be more effective than Illumina sequencing in detecting SVs, which is consistent with Mahmoud’s findings. However, some evidence suggests that short-read data with a high sequencing depth may be able to detect structural variations of the same or greater length than long-read data (Geibel et al.). Several factors may lead to inconsistent results, including species, variation complexity, data accuracy, and software detection efficiency. As long-read sequencing technology matures and becomes more efficient, the detection errors caused by sequencing methods will decrease. PacBio has launched its CCS sequencings, which greatly improves detection accuracy. The emergence of a number of excellent mutation detection software programs has also helped promote mutation research, identifying SVs based on machine learning, like SVFX (Kumar et al., 2020) and dysgu (Cleal and Duncan, 2022). The dysgu software supports the merging of SVs from different callers using different sequencing technologies. It found that the combination of low coverage paired-end and long-reads performs as well as long-reads with higher coverage.

SVs are not distributed uniformly across the genome, as observed in other studies (Liao et al., 2021). The number in each chromosome ranged from 10,148 to 64. The large reference genome tends to have a larger number of SVs, suggesting that each chromosome may have been subjected to different selection pressures during the selection process. Among the different types of SVs, deletions and insertions accounted for the highest proportion, whereas duplications, inversions, and translocations accounted for a smaller proportion (Figure 3). The insertion detection rate was significantly different between long-read and short-read sequencing in this study (Figure 2). Several novel genes have been identified in domestic chickens in a recently published pan-genomic study (Li et al., 2022). To investigate the relationship between insertional SVs in this study and novel genes in chickens, we compared their coordinates with the positions of the identified insertional SVs and found that 4,874 SVs were located in 173 newly discovered genes (Supplementary Table S7). The significance of the new gene for domestic chickens remains to be determined.

### Potential candidate genes affected by structural variants

We identified several genes that were affected by structural variation. They are associated with phenotypes, economic traits, and disease resistance in domestic chicken. Crest is an incomplete dominance mutation that replaces small feathers on the head with dorsal skin feathers (Wang et al., 2012). Li et al. (2021) used short-read re-sequencing to determine that *HOXC10* is related to the crest phenotype in Silkie chickens. Our study also identified this gene mutation in chickens with SK. In contrast to the duplication of SV reported by Li et al., this one is located in an intron with a 19-bp inserted fragment. It is necessary to confirm the results of the mutations affecting this trait. Previously, researchers reported that the expression of *IRX1* and *IRX2* differed between normal and rumpless chickens (Nowlan et al., 2013). Piao chicken is an indigenous Chinese chicken breed lacking a pygostyle, caudal vertebra, uropygial gland, and tail feathers. Our study found a deletion variant (chr2:86914914-86919099) and a duplication variant (2:80868684-87846701) on chromosome two of Piao chicken, the region perches *IRX1* and *IRX2* genes which have been reported to be related to rumplessness in Araucana chickens (Nowlan et al., 2013). This duplication occurred at the lncRNA gene, which is adjacent to *IRX2*. Studies have shown that lncRNAs can regulate the expression of downstream genes by inhibiting the aggregation of ENA polymerase II and inducing chromatin remodeling (Wilusz et al., 2009). In addition, a deletion occurs near *IRX1* in the non-coding region. Non-coding regions in biological genomes account for the majority, and these non-coding regions may have indirect regulatory effects on genes (Alexander et al., 2010). VEP annotation showed that both the SVs were high-impact effector mutations. Hence, we hypothesized that this region might be related to rumpless Piao chickens. We performed PCR to confirm the authenticity of the variant in the subsequent rumplessness of the Piao chicken study. There is still work to be done on the genetic mechanisms of rumplessness in Piao chickens. Our structural variation results also revealed some candidate genes that have been reported to be associated with chicken feather color (*SOX10, CDKN2A*) (Gunnarsson et al., 2011; Schwochow Thalmann et al., 2017), growth (*MYF5, IGF2BP1, CRY1; SH3RF2*) (Jing et al., 2020; Niessner et al., 2011; Krause et al., 2016; Zhang et al., 2020; Wang et al., 2021).

### Application of long-read sequencing in structure variation detection

High-throughput sequencing technology is the mainstream method for SV detection and is considered the gold standard in SV research (Mahmoud et al., 2019; Ho et al., 2020). An increasing number of animal studies have employed long-read sequencing (Long et al., 2018; Bertolotti et al., 2020; Luan et al., 2020). In our study, 49,501 and 22,348 SVs were detected using PacBio and Illumina data, respectively. PacBio sequencing is more sensitive than Illumina data; long-read sequencing can reveal large and complex SV events that are often neglected by short-read sequencing, corroborating the results of a previous study (Liu et al., 2020b). The emergence of long-read sequencing has introduced opportunities and challenges to SVs. More and more studies have showed that SV has the ability to capture the genetic structure differences in breeds and can be used to study population genetic structure. In 2020, researchers carried out structural variation detection in tomatoes using long-read sequencing, and a high-quality pan-genome of SV was constructed, revealing the effects of structural variations on tomato fruit flavor, size, and yield (Alonge, 2020). Weissensteiner (2020) used long-read sequencing to perform SV detection and population evolution in the songbird genera. These results suggest the wealth and evolutionary significance of SV segregation in natural populations. SV combined with selective signal analysis and GWAS analysis is increasing. Because of the limited number of third-generation sequencing samples in this study, it is impossible to carry out population genetic studies based on third-generation sequencing; however, it is believed that SV as a molecular marker will be more widely studied in population genetics in the future.

## Conclusion

SVs are the main source of genomic complexity. We identified chicken SVs using PacBio sequencing. In total, 49,501 SVs were identified across ten chicken breeds. SVs were not evenly distributed in the genomes, and there were several SV-hot sites. During selective breeding of chickens, some SVs that were beneficial to the breed or SVs that had no effect on the genome function of the breed were retained, whereas deleterious SVs were eliminated. Finally, we identified SV-related genes associated with growth, reproduction, and phenotypic appearance that could be artificially selected during chicken domestication.

## Data Availability

The datasets presented in this study can be found in online repositories. The names of the repository/repositories and accession number(s) can be found below: https://www.ncbi.nlm.nih.gov/search/all/?term=PRJNA807790.
