# Supplementary figures and images for: Comprehensive analysis of structural variants in chickens using PacBio sequencing
Source: Front Genet. 2022 Oct 20;13:971588. doi: 10.3389/fgene.2022.971588 (PMC9632285; doi:10.3389/fgene.2022.971588)

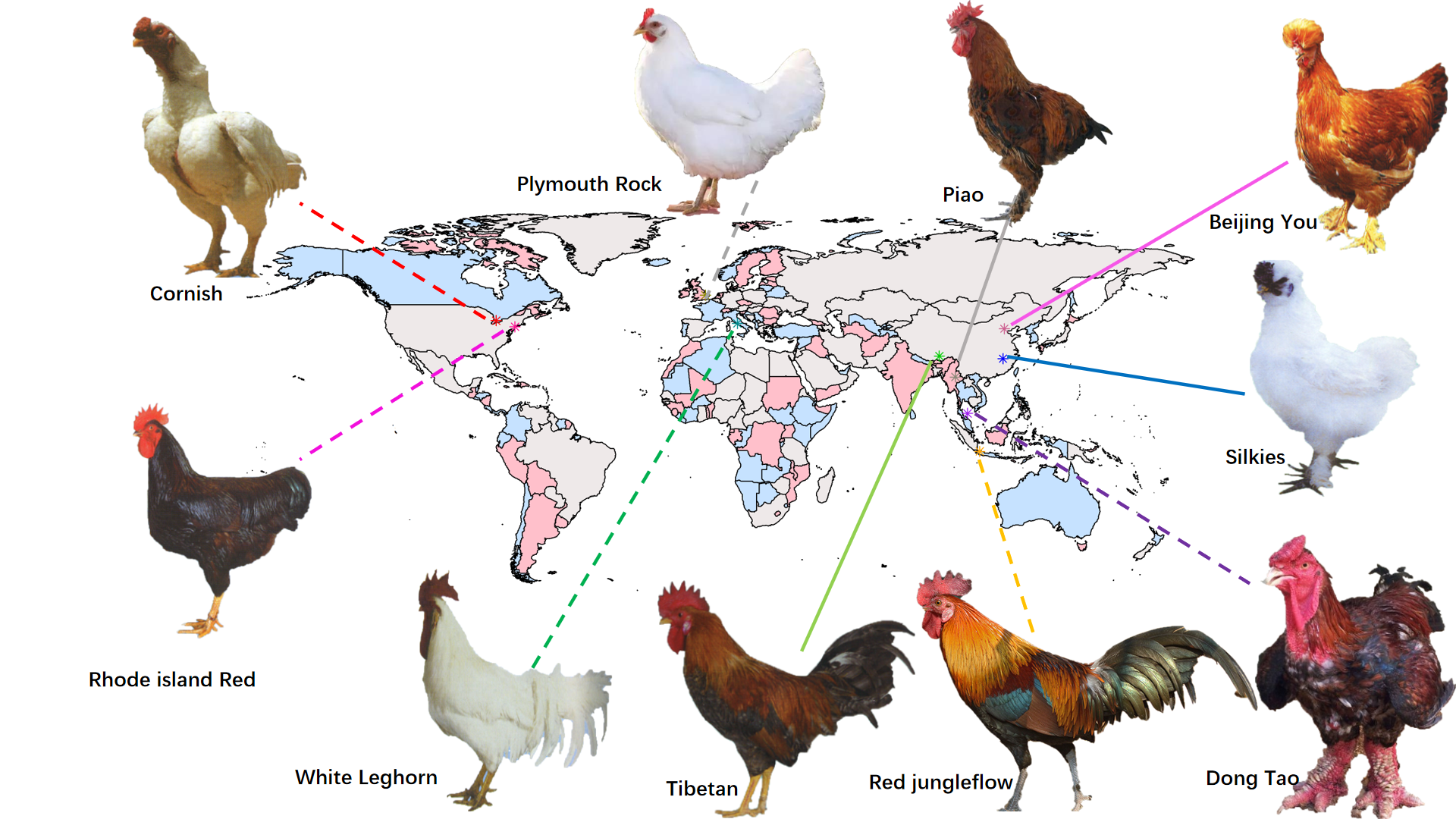

Supplement: Supplementary file 4 [file Image1.TIF]
